# Characterization of the SPI-1 Type III Secretion System in *Pseudomonas fluorescens* 2P24

**DOI:** 10.3389/fmicb.2021.749037

**Published:** 2021-09-21

**Authors:** Jing Wang, Yuan Luo, Yilin Gu, Hai-Lei Wei

**Affiliations:** Key Laboratory of Microbial Resources Collection and Preservation, Ministry of Agriculture and Rural Affairs, Institute of Agricultural Resources and Regional Planning, Chinese Academy of Agricultural Sciences (CAAS), Beijing, China

**Keywords:** *Pseudomonas fluorescens*, PGPR, type III secretion system, transcriptional regulator, ROS

## Abstract

*Pseudomonas fluorescens* 2P24 is a plant growth-promoting rhizobacterium (PGPR) isolated from wheat take-all decline soil. Genomic analysis of strain 2P24 revealed the presence of a complete SPI-1 type III secretion system (T3SS) gene cluster on the chromosome with an organization and orientation similar to the SPI-1 T3SS gene clusters of *Salmonella enterica* and *P. kilonensis* F113. Phylogenetic analysis revealed that the SPI-1 T3SS gene cluster of strain 2P24 might be obtained from *Salmonella* and *Shigella* by horizontal gene transfer. Two transcriptional regulator homologs of HilA and InvF were found from the SPI-1 T3SS gene cluster of strain 2P24. HilA regulated the expression of the structural genes positively, such as *invG*, *sipB*, *sipD*, *prgI*, and *prgK*. Prediction of transcriptional binding sites and RNA-seq analysis revealed 14 genes were up-regulated by InvF in strain 2P24. Exploring potential roles of SPI-1 T3SS revealed that it was not associated with motility. However, 2P24Δ*invF* reduced resistance against *Fusarium graminearum* significantly. 2P24Δ*hilA* enhanced formation of biofilm significantly at 48 h. All three mutants 2P24Δ*hilA*, 2P24Δ*invF*, and 2P24Δ*invE-C* reduced the chemotactic responses to glucose significantly. Finally, the determination of SPI-1 mutants to trigger innate immunity in *Nicotiana benthamiana* showed that 2P24Δ*invE-C* reduced the ability to induce the production of reactive oxygen species compared with the wild type strain 2P24.

## Introduction

Type III secretion system (T3SS) is a protein secretion apparatus that is widespread in animal and plant pathogenic bacteria. T3SS is a syringe-like complex composed of >20 proteins and consists of three parts: a ring component that spans the inner and outer membranes of the bacteria, an extracellular needle/pilus that extends from the outer membrane ring, and a transporter tip that is attached to the host cell membrane (Büttner, 2012; Deng et al., 2017). For adapting to diverse hosts and habitats, T3SS has evolved into seven types: Hrp1, Hrp2, SPI-1, SPI-2, Ysc, RhC, and ChL (Pallen et al., 2005; Troisfontaines and Cornelis, 2005). Among them, Ysc, SPI-1, and SPI-2 T3SS usually exist in animal pathogenic bacteria. Hrp1 and Hrp2 T3SS are mainly present in plant pathogenic bacteria. RhC T3SS is mostly found in plant symbiotic rhizobia. And ChL T3SS often appears in some bacteria that live in animals, insects, and protozoa (Nazir et al., 2017).

Animal and plant pathogens deploy T3SS to deliver type III effectors (T3Es) into host cells for pathogenesis. T3SS structural genes are usually conserved, but T3Es are diverse to adapt to different host environments. The distribution of T3SS and the mechanisms of T3Es in animal and plant pathogens have been studied well. However, the T3SS was also reported to be present in many plant growth-promoting rhizobacteria and especially in the *Pseudomonas fluorescens* group (Preston et al., 2001; Kimbrel et al., 2010; Marchi et al., 2013; Viollet et al., 2017; Stringlis et al., 2019). Preston et al. (2001) found that *P. fluorescens* SBW25 harbored a Hrp1 T3SS, and expression of the *rscC* gene, a *hrcC* homolog, was induced in the sugar beet rhizosphere. Mutations of the *rsp* regulators *rspL* and *rspV* in strain SBW25 resulted in a significant reduction in competitive colonization on the root tips of sugar beet seedlings (Jackson et al., 2005). Genomic analysis of *P. kilonensis* F113 revealed a complete Hrp1 T3SS gene cluster and four T3E homologs RopM, RopAA1, RopAA2, and RopB (Barret et al., 2013). Surprisingly, in addition to the Hrp1 T3SS, SPI-1 T3SS homologs were also found in *P. kilonensis* F113 and some other PGPR, such as *P. fluorescens* HK44 and *P. fluorescens* Q2-87 (Chauhan et al., 2011; Loper et al., 2012; Barret et al., 2013). One report showed that the SPI-1 T3SS of *P. kilonensis* F113 was involved in resistance to amoeboid grazing (Barret et al., 2013).

*Pseudomonas fluorescens* 2P24 is a PGPR isolated from wheat take-all decline soil in Shandong Province, China (Wei et al., 2004a). Strain 2P24 produces several secondary metabolites, such as 2, 4-diacetylphloroglucinol (2, 4-DAPG), hydrogen cyanide (HCN), and siderophore(s), and antagonizes a variety of plant pathogens, such as *Ralstonia solanacearum*, *Rhizoctonia solani*, and *Fusarium oxysporum* (Wei et al., 2004a). Genetic analysis revealed that the antibiotic 2, 4-DAPG is the key biocontrol factor in strain 2P24 (Wei et al., 2004b). In this study, a full length of SPI-1 T3SS gene cluster was identified from strain 2P24. We conducted phylogenetic analysis of the SPI-1 T3SS and transcriptomic analysis of two transcriptional regulators, HilA and InvF. The effects of SPI-I mutation on antagonism, motility, chemotaxis, biofilm formation, and plant immunity were also determined.

## Materials and Methods

### Strains, Plasmids, and Plants

*Escherichia coli* strains were grown in Luria-Bertani (LB) broth at 37°C. *P. fluorescens* was grown in King’s medium B (KB) broth or mannitol-glutamate (MG) minimal medium at 28°C. Pathogenic fungi were grown on Potato Dextrose Agar (PDA) at 28°C, except *Phytophthora*, which was grown on V8 juice agar. Pathogenic bacteria *Ralstonia solanacearum* and *Acidovorax avenae* were grown in Nutrient Broth (NB) at 28°C (Table 1). The following concentrations of antibiotics were used: ampicillin at 50 μg/mL and kanamycin at 50 μg/mL. *Nicotiana benthamiana* was grown in a greenhouse with 16 h light/8 h dark, 65% humidity, and temperatures of 24°C during daylight and 22°C at night.

**TABLE 1:** Strains and plasmids used in this study.

**Strains or plasmids**	**Characteristics**	**Sources**
**Strains**		
*P. fluorescens* 2P24	Ap^*r*^, wild type, PGPR	Wei et al., 2004a
*P. fluorescens* 2P24Δ*hilA*	Ap^*r*^, strain 2P24 deleted *hilA* fragment	This study
*P. fluorescens* 2P24Δ*invF*	Ap^*r*^, strain 2P24 deleted *invF* fragment	This study
*P. fluorescens* 2P24Δ*invE-C*	Ap^*r*^, strain 2P24 deleted *invE*, *invA*, and *invC* fragments	This study
*P. fluorescens* Pf0-1	Ap^*r*^, wild type, non-T3SS bacterium	Compeau et al., 1988
*Rhizoctonia solani*	Pathogenic fungus	Lab collection
*Fusarium graminearum*	Pathogenic fungus	Lab collection
*Magnaporthe oryzae*	Pathogenic fungus	Lab collection
*Phytophthora nicotianae*	Pathogenic fungus	Lab collection
*Ralstonia solanacearum* GMI1000	Pathogenic bacteria	Lab collection
*Acidovorax avenae*	Pathogenic bacteria	Lab collection
*Escherichia coli* DH5α	F^–^φ80 *lacZ*ΔM15Δ(*lacZYA*-*argF*) U169 *endA1 recA1 hsdR17* (r_*k*_^–^, m_*k*_^+^) *supE44*λ^–^ *thi*-1 *gyrA96 relA1 phoA*	Sambrook et al., 1989
**Plasmids**		
pK303SacB	Km^*r*^, *oriT*^+^ *sacB*^+^	Lab collection
pK303SacBΔ*hilA*	Km^*r*^, pK303SacB containing the left and right flanking regions of *hilA* gene	This study
pK303SacBΔ*invF*	Km^*r*^, pK303SacB containing the left and right flanking regions of *invF* gene	This study
pK303SacBΔ*invE-C*	Km^*r*^, pK303SacB containing the left and right flanking regions of *invE-C* genes	This study
pRK2013	Km^*r*^, ColE1 replicon with RK2 transfer region, helper plasmid	Figurski and Helinski, 1979

### Bioinformatic Analyses

To locate the SPI-1 T3SS gene cluster, we performed a BlastP search in the complete genome of strain 2P24 (GenBank accession: CP025542.1), using protein sequences of T3SS conserved components that were identified previously in *Salmonella enterica* (InvA: QBG67165.1, SpaP: EBB2042894.1, and PrgK: NUD93968.1). DNAMAN Version 9 was used to analyze the identity of T3SS components between *P. fluorescens* 2P24, *P. kilonensis* F113, and *S. enterica*. To produce a phylogenetic analysis of SPI-1, we searched the National Center of Biotechnology Information (NCBI) database for InvA protein sequences and *16S rRNA* sequences in five genera: *Pseudomonas*, *Salmonella*, *Shigella*, *Yersinia*, and *Burkholderia*. Phylogenetic trees were created with MEGA7 (Kumar et al., 2016) using the Maximum Likelihood method.

For scanning InvF binding sites from intergenic regions of strain 2P24, Geneious Prime^®^ (Biomatters Ltd) was used to generate intergenic regions by retrieving the genome sequence of strain 2P24. Then, we input the sequence matrix of the InvF binding site ‘‘ANNGGNCNTTTTTTNAANGTT’’ and the intergenic region file to the Find Individual Motif Occurrences (FIMO)^1^ (Grant et al., 2011); we set the *p* value ≤ 10^–5^.

### Construction of the *hilA*, *invF*, and *invE-C* Mutants

To make a *hilA* mutant of *P. fluorescens* 2P24, a 2.8 kb fragment that covered the full length of the *hilA* gene was amplified from strain 2P24 using primer pairs WHL140 (5′-TTCTAAGCTTGAGCAACAGCAGCG-3′) and WHL141 (5′-GGACACGCCACTTCTAGAAGTTGG-3′) and digested with *Hin*dIII and *Xba*I. The fragment was cloned into the same sites of pK303SacB. The resulting pK303SacB derivative was digested with *Nar*I to eliminate a 0.9 kb fragment of the *hilA* gene from the insertion. The parental fragment was recycled and self-ligated. The final pK303SacB derivative pK303SacBΔ*hilA* was constructed and transformed into strain 2P24. Colonies were selected from KB medium with 10% sucrose. Then kanamycin-sensitive mutants were selected and screened by PCR.

To make an *invF* mutant of strain 2P24, two 1.0 kb fragments that carried the left and right flanking regions of *invF* were amplified by PCR using primer pairs WHL92 (5′-CCAAAGCTTCAGGACTGGTCACGCC-3′) and WHL93 (5′-GGCATCGCTCATGTCGAAAAAAGC-3′), and primer pairs WHL94 (5′-CTCGCTGACCGATGTGGCACT-3′) and WHL95 (5′-TCGAGGATCCACAACGACAACTC-3′), respectively. The left and right regions were digested with *Hin*dIII and *Bam*HI, respectively, and were cloned into the relevant sites of pK303SacB. The resulting pK303SacB derivative pK303SacBΔ*invF* was transformed into strain 2P24. The correct mutant was screened using the same method as *hilA* mutant, and the same protocol was used to make an *invE-C* mutant. The left and right flanking regions of *invE-C* were amplified by PCR using primer pairs WHL15 (5′- TGGCGAATTCATCCTCGGAACAAG-3′) and WHL16 (5′- CTGACAATTCGTCGCTGATCTGC-3′), and primer pairs WHL17 (5′-GCGTCTCGAGCAACTGCAGGTCT-3′) and WHL18 (5′-ATCAAGCTTGTATCGGCAGCGGT-3′), respectively. The left and right regions were digested with *Eco*RI and *Hin*dIII, respectively, and then cloned into the relevant sites of pK303SacB.

### RNA Preparation

Strains 2P24, 2P24Δ*invF*, and 2P24Δ*hilA* were grown on KB plates with 50 μg/mL ampicillin at 28°C. Fresh lawns were suspended in 5 mL of KB and incubated at 28°C overnight with 200 rpm shaking. Bacterial suspension was diluted in 50 mL of MG medium in flasks to a final OD_600_ of 0.1. The cultures were incubated at 28°C at 200 rpm on a rotary shaker for 6 h/12 h. The cell fractions were separated by centrifugation at 10, 000 rpm for 5 min at 4°C. Total RNA was isolated using a E.Z.N.A.^®^ Bacterial RNA Kit (Omega Bio-tek, United States) as described by the manufacturer for RNA-seq and qRT-PCR.

### RNA-Seq and qRT-PCR

A prokaryotic chain specific sequencing library was constructed using a NEBNext Ultra Directional RNA Library Prep Kit and then sequenced on an Illumina HiSeq platform. Gene expression was calculated using HTseq software (V 0.6.1) based on the FPKM (Expected number of Fragments Per Kilobase of transcript sequence Per Millions base pairs sequenced) method. Genetic differences were analyzed using DESEQ2 (V1.6.3) in the Bioconductor software package. For qRT-PCR, a FastKing RT Kit (With gDNase) (Tiangen, China) was used to synthesize cDNA from 50 ng-2 μg of total RNA. Luna Universal qPCR Master Mix (New England Biolabs, United States) was used for a real time PCR reaction to quantify the cDNA level of target genes in different samples. Reactions were run and data were collected using the ABI QuantStudio6 Flex real-time PCR system (Applied Biosystems, United States).

### *In vitro* Tests of Pathogenic Antagonism

Antagonistic tests were performed as described by Gu et al. (2020). For antagonism of pathogenic fungi, a 1 cm diameter agar plug with mycelium was placed in the center of an agar plate, and 10 μL cultures of strains 2P24, 2P24Δ*hilA*, 2P24Δ*invF*, and 2P24Δ*invE-C* were dropped on the plate at four directions, approximately 3 cm from the center. The plates were incubated at 28°C and checked for inhibition zones of mycelial growth. For antagonism of pathogenic bacteria, we incubated pathogenic bacteria in 5 mL of NB overnight and diluted in melting Nutrient Agar (NA) at a ratio of 1:100 for preparing plates. The cultures of strains 2P24, 2P24Δ*hilA*, 2P24Δ*invF*, and 2P24Δ*invE-C* were inoculated and observed as above.

### Motility Tests

We conducted motility tests as described by Rashid and Kornberg (2000), with slight modification. For swimming tests, we used KB medium that contained 0.2% agar, and for swarming tests, we used KB medium that contained 0.5% agar and 0.5% glucose. Freshly cultured strains were dipped using pipette tips and inoculated on the surface of the plates’ center. For twitching tests, we used KB medium that contained 1% agar. Freshly cultured strains were dipped using pipette tips and inoculated into the bottom of the plates’ center. Then, plates were placed stably in the incubator and cultured at 28°C.

### The Chemotactic Detection of Glucose

Chemotactic responses of strain 2P24 and SPI-1 related mutants to glucose were performed according to a simplified capillary assay method (Mazumder et al., 1999). Bacteria grew to late log phase in KB liquid medium. Pellets were collected by centrifugation and washed twice with BHA (NH_4_NO_3_, 1 g/L; FeCl_3_, 0.05 g/L; KH_2_PO_4_, 1 g/L; K_2_HPO_4_, 1 g/L; MgSO_4_, 0.2 g/L; CaCl_2_, 0.02 g/L; pH 7.0) (Ugochukwu et al., 2013). Finally, bacteria were suspended into BHA (OD_600_ = 1.0). One hundred microliters of bacterial suspension were sucked into a 200 μL pipette tip, and 100 μL BHA that contained 0.1% glucose was drawn up through the needle into a 1 mL syringe, and 100 μL BHA without carbon source was drawn up through the needle into a 1 mL syringe as negative control. The needle-syringe capillary was inserted into the pipette tip that contained the bacterial suspension. After 45 min of incubation at room temperature, the contents from the needle-syringe were removed and diluted in 25 mM PBS buffer (pH 7.0) and plated onto LB medium. Accumulation in the capillary was calculated from the CFUs on the plates.

### Biofilm Formation Test

The assay of biofilm formation was performed according to the classic approach (Christensen et al., 1982). One hundred microliters cultures of bacterial strains that were incubated overnight were added to 2 mL centrifuge tubes, and 900 μL KB was added to each tube. Bacterial strains were incubated at 28°C for 48 h statically. Then, the contents of each tube were emptied and washed three times with sterile water. The tubes were stained for 15 min with 200 μL of 1% crystal violet and then washed with sterile water. After the tubes were air dried, the dye bound to the adherent cells was resolubilized with 2 mL of 95% ethyl alcohol per tube. The OD of each tube was measured at 570 nm by using a L3S visible spectrophotometer (INESA, China).

### Assay for Reactive Oxygen Species

For ROS assays, bacterial strains were injected into 5-wk-old *N. benthamiana* leaves at 5 × 10^8^ cfu/mL. Leaf disks were excised 12 h later with a 0.5 cm diameter cork borer and placed into wells of 96-well plates to which 10 μL ddH_2_O had been pre-added. Then, 100 μL of 0.5 mM L-012 (Wako, Kyoto, Japan) in 10 mM morpholinepropanesulfonic acid-KOH buffer (pH 7.4) was added. Chemiluminescence was monitored immediately for 10 h using a Veritas luminometer (GENios Pro, TECAN, Switzerland).

### Statistical Analyses

All tests in this study were performed at least triplicate, and standard deviations were calculated. One-way ANOVA was performed to determine significant changes.

## Results

### Characterization of the SPI-1 T3SS Components in *Pseudomonas fluorescens* 2P24

**FIGURE 1:**
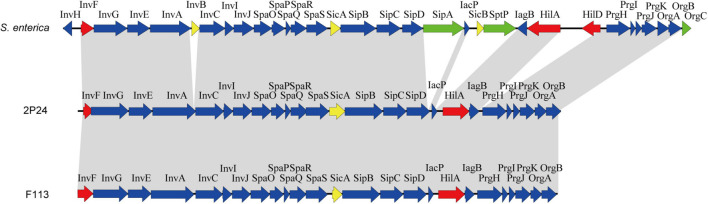
Genetic organization of the SPI-1 T3SS cluster of *P. fluorescens* 2P24 and comparison with the clusters of *Salmonella enterica* and *P. kilonensis* F113. Predicted open reading frames and their orientation are shown by large arrows. T3SS accessory protein encoding genes are shown as blue arrows, transcriptional regulator encoding genes are shown as red arrows, effector protein encoding genes are shown as green arrows, and chaperone protein encoding genes are shown as yellow arrows. Homologous genes are connected with gray shading.

Through genome mining and comparison, a 23 kb SPI-1 T3SS gene cluster comprised of 25 predicted open reading frames (ORFs) was identified in *P. fluorescens* 2P24, and the gene names were assigned according to the SPI-1 T3SS of *S. enterica*. Comparing SPI-1 T3SS gene clusters of PGPR and mammalian pathogens indicated that the organization and orientation of SPI-1 T3SS in strain 2P24 were very close to those in *S. enterica* and *P. kilonensis* F113 (Figure 1). The SPI-1 T3SS of strain 2P24 retained all assembly proteins except InvH, which indicated that the structure of SPI-1 T3SS in strain 2P24 was conserved. Two transcriptional regulator homologs, HilA and InvF, were retained in strain 2P24. However, compared with the SPI-1 T3SS in *S. enterica*, effector proteins such as SipA, SptP, and OrgC were lost, and only the chaperone protein SicA was retained in strains 2P24 and F113. Amino acid sequence analysis of the SPI-1 T3SS components in strain 2P24 showed higher identity to strain F113 than to *Salmonella* (Table 2). The export apparatus (i.e., InvA, SpaP, SpaQ, SpaR, and SpaS), basal body proteins (InvG and PrgK), accessory protein (IagB), needle (PrgI), and chaperone protein (SicA) displayed >50% identity between *S. enterica* and strain 2P24, although the remaining accessory proteins (i.e., InvE, InvC, InvI, SpaO, PrgH, PrgJ, OrgA, and OrgB) showed less identity (between 25 and 50%). In addition, less conserved regulators and translocons suggested a changed function of SPI-1 T3SS in PGPR.

**TABLE 2:** Comparison of SPI-1 T3SS cluster proteins in *P. fluorescens* group and *S. enterica*.

		**Length of predicted peptides (a.a.) in the following**		
		**strains/percentage identity to that of strain 2P24**		
	**Length of predicted**			
**ORFs**	**peptides (a.a.) in strain 2P24**	***S. enterica***	**F113**	**Predicted function**
*invF*	163	216/44	245/58	Transcriptional regulator
*invG*	590	562/53	549/83	Basal body, OM ring
*invE*	368	372/47	368/90	Export apparatus, gate keeper
*invA*	683	685/65	683/95	Export apparatus, gate protein
*invC*	446	431/46	428/87	Export apparatus, ATPase
*invI*	151	147/26	151/88	Export apparatus, central stalk
*invJ*	297	336/16	294/58	Needle length regulator
*spaO*	307	303/32	307/73	Export apparatus, C-ring homolog
*spaP*	219	224/64	219/94	Export apparatus, IM component
*spaQ*	84	86/66	84/96	Export apparatus, IM component
*spaR*	267	263/53	267/92	Export apparatus, IM component
*spaS*	367	356/58	344/90	Export apparatus, autoprotease
*sicA*	250	165/57	160/62	Chaperone protein
*sipB*	595	593/27	589/88	Translocon, minor subunit
*sipC*	365	409/12	363/72	Translocon, major subunit
*sipD*	368	343/38	365/61	Tip, tip protein
*iacP*	85	82/24	84/70	Translocation regulator
*hilA*	419	553/36	419/90	Transcriptional activator
*iagB*	155	160/54	154/84	Accessory protein, lytic transglycosylase
*prgH*	378	392/39	391/76	Basal body, IM ring
*prgI*	86	80/51	87/82	Needle, filament protein
*prgJ*	102	101/37	102/94	Basal body, inner rod
*prgK*	240	252/56	239/88	Basal body, IM ring
*orgA*	188	199/27	188/80	Export apparatus, cytoplasmic protein
*orgB*	235	223/26	235/72	Export apparatus, peripheral stalk

*OM, outer membrane; IM, inner membrane.*

### Phylogenetic Analysis of SPI-1 T3SS Among Beneficial and Pathogenic Bacteria

**FIGURE 2:**
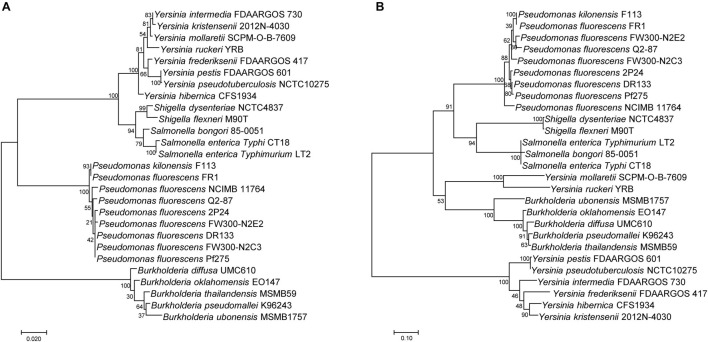
Phylogenetic analysis of SPI-1 T3SS gene clusters of the *P. fluorescens* group. **(A)** A distance tree was calculated from *16S rRNA* homologs by using the Maximum Likelihood method based on the Tamura-Nei model. **(B)** A distance tree was calculated from InvA homologs by using the Maximum Likelihood method based on the JTT matrix-based model.

A few reports showed that the SPI-1 T3SS was present in some plant beneficial bacteria, especially in fluorescent *Pseudomonas* (Chauhan et al., 2011; Loper et al., 2012). To investigate the relationship of SPI-1 T3SS among beneficial and pathogenic bacteria, the *16S rRNA* sequences and InvA amino acid sequences were compared among *Pseudomonas*, *Salmonella*, *Shigella*, *Yersinia*, and *Burkholderia* spp. Based on alignment of the *16S rRNA*, the maximum likelihood method was used to construct a phylogenetic tree that depicted evolutionary distance among these T3SS-containing bacteria (Figure 2A). There were two clades in the tree, and all of the fluorescent *Pseudomonas* strains were located on a separate subbranch of one clade, which indicated a distant evolutionary relationship with mammalian pathogens. Then, the SPI-1 T3SS phylogenetic tree was constructed based on InvA amino acid sequences using the same method (Figure 2B). The phylogenetic tree contained two clades, and fluorescent *Pseudomonas* strains were located in a subbranch together with *Shigella* spp. and *Salmonella* spp. The difference in phylogenetic relationship between *16S rRNA* and InvA suggested a horizontal transfer of SPI-1 T3SS from mammalian pathogens, such as *Salmonella* and *Shigella*, to plant beneficial fluorescent *Pseudomonas*.

### HilA Up-Regulates the Structural Genes of SPI-1 T3SS

**FIGURE 3:**
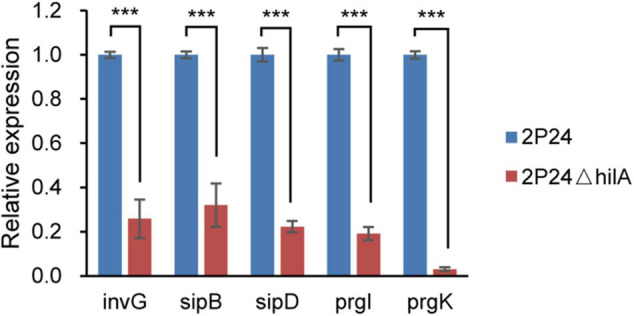
Expression profiles of *invG*, *sipB*, *sipD*, *prgI*, and *prgK* in strains 2P24 and 2P24Δ*hilA* by quantitative reverse transcriptase-polymerase chain reaction (qRT-PCR). Graph shows gene expression levels in strains 2P24 or 2P24Δ*hilA* at 12 h after being incubated in MG medium, as quantified by qRT-PCR. Gene expression was normalized to the expression level of reference gene *recA* using the 2^–ΔΔ*Ct*^ method. Data are means of three replicates. Error bars represent SD. *** represents *P* < 0.001.

HilA is a transcriptional activator, which regulates transcription of the SPI-1 T3SS apparatus genes directly (Ellermeier and Slauch, 2007). To verify whether HilA had a similar function in strain 2P24, the *hilA* deficient mutant 2P24Δ*hilA* was constructed, and the expression of five apparatus genes (*invG*, *sipB*, *sipD*, *prgI*, and *prgK*) of the SPI-1 T3SS complex in strains 2P24 and 2P24Δ*hilA* was detected by qRT-PCR. Compared to strain 2P24, the expression of these five apparatus genes was decreased significantly in 2P24Δ*hilA* (Figure 3). HilA of strain 2P24 had a transcriptional activation function similar to mammalian pathogens and regulated SPI-1 apparatus genes positively.

### Identification of the Regulon of Transcriptional Regulator InvF

**FIGURE 4:**
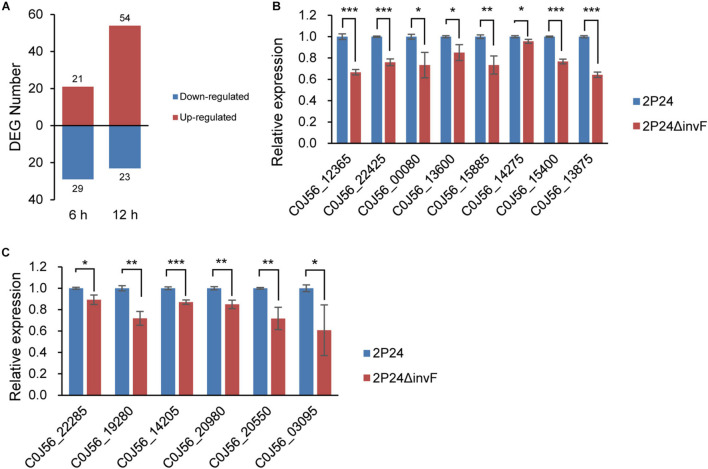
Identification of InvF regulons. **(A)** Number of up-regulated (red bars) and down-regulated (blue bars) differentially expressed genes (DEGs) from RNA-seq analysis of 2P24Δ*invF* compared with strain 2P24 at 6 and 12 h after being incubated in MG medium [Fold change ≥ 1.5; *p* value ≤ 0.05]. **(B)** Expression profiles of InvF up-regulated genes selected from RNA-seq DEGs in strains 2P24 and 2P24Δ*invF* by qRT-PCR. Graph shows gene expression levels in strains 2P24 or 2P24Δ*invF* at 6 or 12 h after being incubated in MG medium, as quantified by qRT-PCR. **(C)** Expression profiles of InvF up-regulated genes selected from the adjacent downstream genes of predicted InvF binding sites in strains 2P24 and 2P24Δ*invF* by qRT-PCR. Graph shows gene expression levels in strains 2P24 or 2P24Δ*invF* at 6 h after being incubated in MG medium, as quantified by qRT-PCR. Gene expression was normalized to the expression level of reference gene *recA* using the 2^–ΔΔ*Ct*^ method. Data are means of three replicates. Error bars represent SD. *, **, and *** represent *P* < 0.05, *P* < 0.01, and *P* < 0.001, respectively.

InvF, an AraC-type transcriptional activator, regulated the expression of genes that encoded the secreted effector molecules SipABCD, SigD, SptP, and SopE (Darwin and Miller, 1999; 2001). However, it seems that almost all effector homologs were lost in strain 2P24 (Figure 1). To identify the InvF regulon in strain 2P24, the *invF* deficient mutant 2P24Δ*invF* was constructed, and we implemented transcriptome sequencing (GEO accession: GSE181272). A total of 127 significantly differentially expressed genes (DEGs) were obtained by comparing the transcriptional level of strain 2P24Δ*invF* with 2P24 at 6 h and 12 h [Fold change ≥ 1.5; *p* value ≤ 0.05] (Figure 4A and Supplementary Table 1). KEGG analyses indicated that DEGs at 6 h and 12 h involved biosynthesis of secondary metabolites, biosynthesis of amino acids, and ABC transporters (Supplementary Figure 1). Fifty-two down-regulated genes were regarded as InvF regulon candidates. Among the InvF regulon candidates, 39 genes were selected for qRT-PCR verification and eight genes were confirmed to be regulated positively by InvF (Figure 4B).

Previous study found an InvF binding site in the promoter regions of *sicA*, *sigD*, and *sopE* that was recognized by InvF to activate downstream expression of genes (Darwin and Miller, 2001). To identify the InvF regulon more comprehensively, the intergenic regions of the genome of strain 2P24 were scanned for the InvF binding site using the FIMO website (see text footnote 1) (Grant et al., 2011). Eleven InvF binding sites were obtained at *p* value ≤ 1 × 10^–5^ (Supplementary Table 2). The adjacent downstream genes of these binding sites were searched, and their expression in strains 2P24 and 2P24Δ*invF* was verified by qRT-PCR (Supplementary Table 2). Six genes among them were regulated positively by InvF (Figure 4C). Finally, 14 genes were identified as InvF regulons through transcriptional and promoter analysis (Table 3). However, all of the InvF regulons are not homologous to any effector proteins in pathogenic bacteria.

**TABLE 3:** InvF regulons up-regulated by InvF and the predicted information about signal peptide of each protein sequence.

**Gene_ID**	**Product**	**Signal peptide**	**Screening approach**
C0J56_12365	Dihydrolipoyl dehydrogenase	None	Transcriptome analysis
C0J56_22425	Toxin-activating lysine-acyltransferase	None	Transcriptome analysis
C0J56_00080	Phage infection protein	Signal peptide (Sec/SPI)	Transcriptome analysis
C0J56_13600	AsnC family protein	None	Transcriptome analysis
C0J56_15885	Putative porin	Signal peptide (Sec/SPI)	Transcriptome analysis
C0J56_14275	Fap system putative outer membrane protein	Signal peptide (Sec/SPI)	Transcriptome analysis
C0J56_15400	NAD(P)H dehydrogenase	None	Transcriptome analysis
C0J56_13875	Protein iolH	None	Transcriptome analysis
C0J56_22285	DNA-binding response regulator	None	Promoter analysis
C0J56_19280	SDR family oxidoreductase	None	Promoter analysis
C0J56_14205	LEA type 2 family protein	Lipoprotein signal peptide (Sec/SPII)	Promoter analysis
C0J56_20980	23S rRNA pseudouridine (955/2504/2580) synthase RluC	None	Promoter analysis
C0J56_20550	Bacteriocin homologous protein	Signal peptide (Sec/SPI)	Promoter analysis
C0J56_03095	Hypothetical protein	None	Promoter analysis

### InvF Partially Affects Antagonism of Strain 2P24 Against *Fusarium graminearum*

**FIGURE 5:**
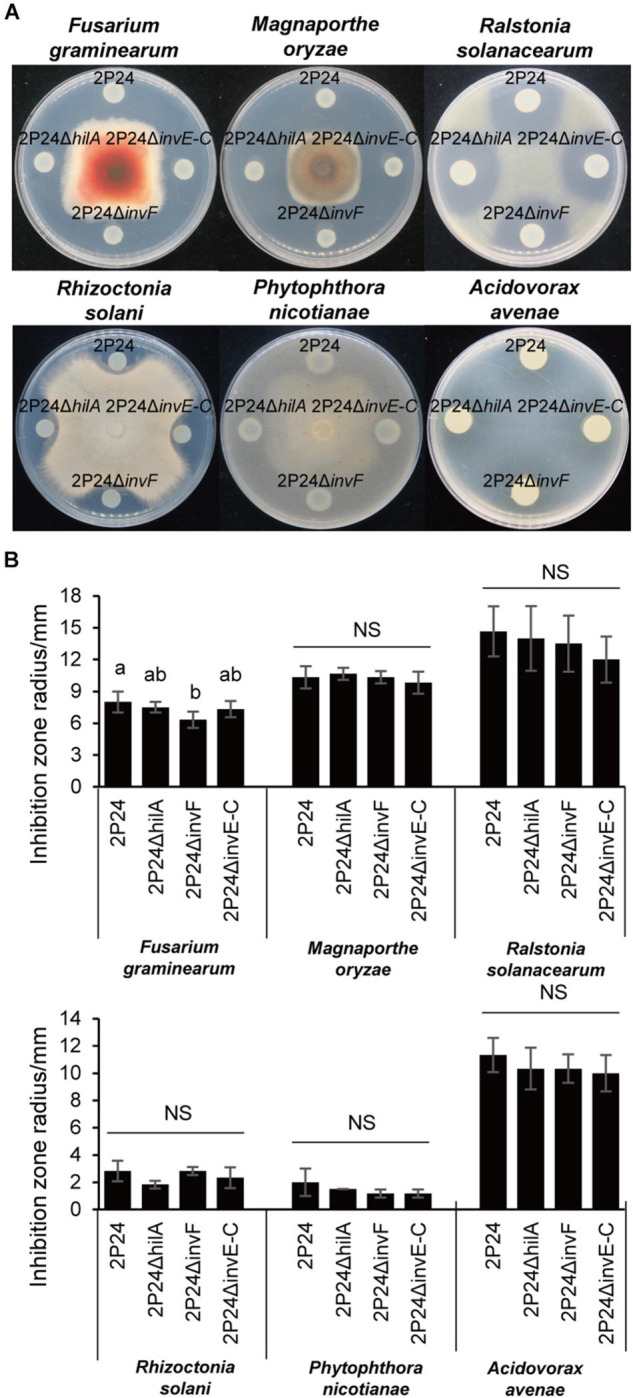
Antagonism of strain 2P24 and SPI-1 related mutants to plant pathogens. **(A)** Antagonistic effect on pathogenic fungi *Fusarium graminearum*, *Magnaporthe oryzae*, *Rhizoctonia solani*, and *Phytophthora nicotianae* and pathogenic bacteria *Ralstonia solanacearum* and *Acidovorax avenae*. **(B)** Quantitative analysis of antagonistic effect. Data are means of three replicates. Error bars represent SD. Means shown with the same letters are not different statistically at the 5% confidence level on the basis of Duncan’s multiple range test. NS means no significant difference.

A previous study found that strain 2P24 antagonized multiple plant pathogens (Wei et al., 2004a). To investigate whether SPI-1 T3SS was involved in the antagonism, the T3SS deficient mutant 2P24Δ*invE-C* was constructed. Then, the antagonistic ability of strains 2P24, 2P24Δ*hilA*, 2P24Δ*invF*, and 2P24Δ*invE-C* against various plant pathogens was detected. 2P24Δ*invE-C* and 2P24Δ*hilA* exhibited no significant difference with strain 2P24 in antagonism (Figure 5). 2P24Δ*invF* also showed no significant difference with strain 2P24 in antagonism to a variety of pathogens, but it reduced antagonistic ability against *F. graminearum* significantly. We speculated that InvF might influence antagonism to *F. graminearum* indirectly by regulating the expression of some genes rather than the T3SS.

### SPI-1 T3SS Is Not Associated With Motility but Involved in Chemotaxis of Strain 2P24

**FIGURE 6:**
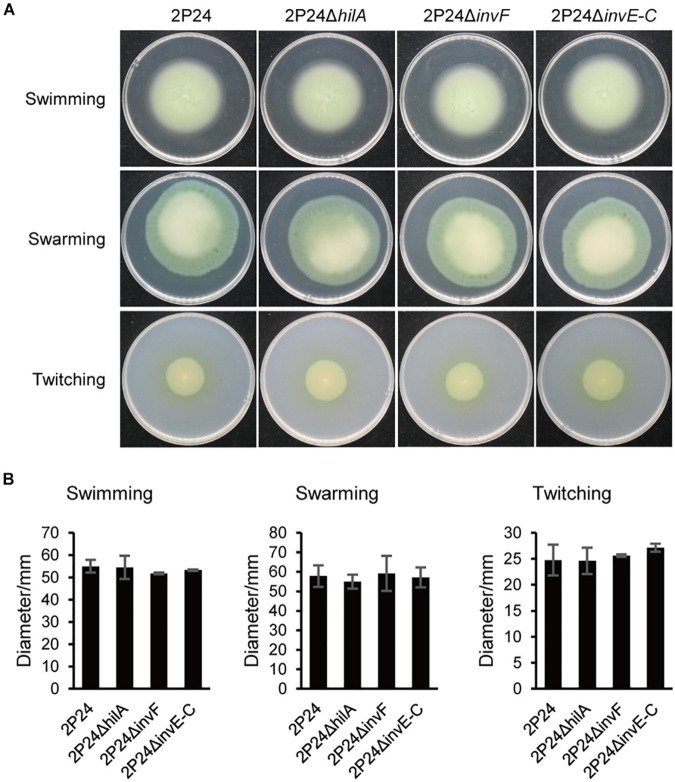
Motility of strain 2P24 and SPI-1 related mutants. **(A)** Swimming on the 0.2% agar-contained KB medium for 42 h, swarming on the 0.5% agar-contained KB medium for 48 h, and twitching in the 1% agar-contained KB medium for 96 h. **(B)** Quantitative analysis of swimming, swarming, and twitching of strain 2P24 and SPI-1 related mutants. Data are means of three replicates. Error bars represent SD.

Bacteria can carry out single bacterial swimming and multiple bacterial swarming by the rotational movement of flagella. In addition, the expansion and contraction of type IV secretion system (T4SS) can drive twitching (Henrichsen, 1972). To explore the influence of SPI-1 T3SS in the motility of strain 2P24, we detected swimming, swarming, and twitching of strain 2P24 and the SPI-1 mutants. Strains 2P24, 2P24Δ*hilA*, 2P24Δ*invF*, and 2P24Δ*invE-C* exhibited similar swimming, swarming, and twitching motility under different concentrations of agar (Figure 6A). Statistical analysis showed that no significant difference was found between the strains (Figure 6B).

**FIGURE 7:**
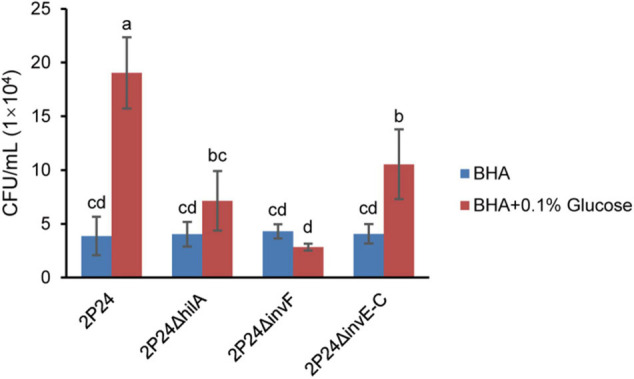
The chemotactic responses of strain 2P24 and SPI-1 related mutants to glucose. Data are means of three replicates. Error bars represent SD. Means shown with the same letters are not different statistically at the 5% confidence level on the basis of Duncan’s multiple range test.

Bacteria perceive the change of chemical concentration in the environment to produce an approach or a retreat response. This chemotactic behavior usually depends on the motility of flagella and can help bacteria survive better (Chet and Mitchell, 1976). In this study, we tested the influence of SPI-1 T3SS in perceiving exogenous glucose. Strain 2P24 had a strong chemotactic response to glucose. However, 2P24Δ*hilA*, 2P24Δ*invF*, and 2P24Δ*invE-C* reduced the chemotactic response to glucose significantly (Figure 7). These results suggest that the SPI-1 T3SS of strain 2P24 is involved in chemotaxis independent on motility.

### 2P24Δ*hilA* Enhances Biofilm Formation

**FIGURE 8:**
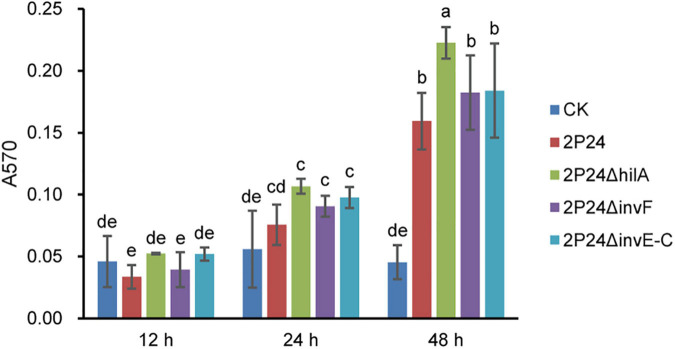
Biofilm formation of strain 2P24 and SPI-1 related mutants incubated in KB medium for 12, 24, and 48 h in 2 mL centrifuge tubes. Data are means of three replicates. Error bars represent SD. Means shown with the same letters are not different statistically at the 5% confidence level on the basis of Duncan’s multiple range test.

Biofilm formation of bacteria is related to many factors. Ding and Wang (2009) reported that the T3SS mutant of *Yersinia pseudotuberculosis* reduced its ability to form biofilm significantly. To verify whether SPI-1 T3SS affected the ability of strain 2P24 to form biofilm, we detected the biofilm of strain 2P24 and related mutants of SPI-1 after static incubation at 28°C for 12, 24, and 48 h in 2 mL centrifuge tubes. Compared with negative control, strain 2P24 formed obvious biofilm at 48 h (Figure 8). 2P24Δ*invF* and 2P24Δ*invE-C* showed no significant difference with wild type, but 2P24Δ*hilA* enhanced biofilm formation significantly (Figure 8) which suggest that HilA plays important role in biofilm formation via a transcriptional regulation on some genes. However, the mechanism remains to be explored further.

### 2P24Δ*invE-C* Reduces the Activation of ROS Burst in *Nicotiana benthamiana*

**FIGURE 9:**
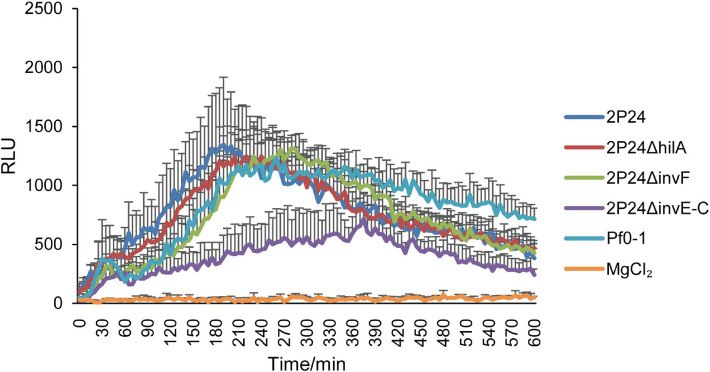
ROS assay of strain 2P24 and SPI-1 related mutants. The bacteria were infiltrated at 5 × 10^8^ cfu/mL into *N. benthamiana* leaves and 12 h later assayed for ROS burst using L-012 chemiluminescence. *P. fluorescens* Pf0-1 was used as the positive control and 10 mM MgCl_2_ as the negative control. Data are means of six replicates. Error bars represent SD.

Previous study found that strain 2P24 triggered a ROS burst in *N. benthamiana* leaves (Liu et al., 2016). In this study, we detected whether SPI-1 T3SS was involved in triggering a ROS burst in *N. benthamiana*. After injection of *N. benthamiana* with wild type and SPI-1 mutants of strain 2P24 for 12 h, ROS was measured every 5 min for 10 h. Consistent with *P. fluorescens* Pf0-1, strain 2P24 triggered a strong ROS burst, and deficient mutants of *hilA* and *invF* did not influence this immunity (Figure 9). However, 2P24Δ*invE-C* reduced the accumulation of ROS significantly (Figure 9). This indicated that the SPI-1 T3SS was involved in triggering a ROS burst in *N. benthamiana*.

## Discussion

The Salmonella Pathogenicity Island 1 (SPI-1) family T3SS mainly exists in mammalian pathogens as a pathogenic apparatus. Recently, SPI-1 T3SS was also found in plant-associated bacteria such as *Xanthomonas* (Alavi et al., 2008; Marguerettaz et al., 2011), *Erwinia* (Triplett et al., 2006), *Pantoea* (Correa et al., 2012; Kirzinger et al., 2015), and *Pseudomonas* species (Barret et al., 2013; Redondo-Nieto et al., 2013). However, the function of SPI-1 T3SS in these plant bacteria is unclear. In this study, SPI-1 T3SS of PGPR *P. fluorescens* 2P24 was characterized, and the function was determined.

A 23 kb SPI-1 T3SS gene cluster was found in the chromosome of strain 2P24, and its organization and orientation were similar to that in *S. enterica* (Figure 1). This cluster consisted of 25 ORFs encoding structural and regulatory proteins of T3SS, but it had lost almost all of the effector encoding genes (Figure 1 and Table 2). Previous studies have found that beneficial fluorescent *Pseudomonas* that contained Hrp1 T3SS also lost most of its effector homologs (Jackson et al., 2005; Kimbrel et al., 2010; Marchi et al., 2013; Stringlis et al., 2019). This phenomenon suggests that the function of T3SS in plant beneficial *Pseudomonas* may have changed compared to pathogens. PGPR strains live in the rhizosphere of plants, relying on the interaction with the surrounding environmental factors, and do not need to transport proteins into host cells. This might result in the loss of effector homologs in *P. fluorescens* 2P24.

To explore genetic evolution of SPI-1 T3SS further in fluorescent *Pseudomonas*, phylogenetic trees of *16S rRNA* and InvA in *Pseudomonas*, *Salmonella*, *Shigella*, *Yersinia*, and *Burkholderia* spp. were constructed (Figure 2). The results suggested that SPI-1 T3SS in fluorescent *Pseudomonas* might be obtained from *Salmonella* and *Shigella* by horizontal gene transfer. A previous study also found that there was horizontal gene transfer of SPI-1 T3SS in *Pantoea* by analyzing its phylogenetic relationship and GC content (Kirzinger et al., 2015). The increase in recent genome sequences has shown that the homologs of SPI-1 T3SS are present in many other bacterial species, such as *Sodalis*, *Erwinia*, *Pantoea*, *Pandoraea*, *Pectobacterium*, *Providencia*, and *Serratia* (Egan et al., 2014). A more systematic evolutionary analysis of SPI-1 T3SS is needed.

The SPI-1 T3SS of strain 2P24 retained two transcription factor homologs, HilA and InvF. HilA performed a similar transcriptional activation function to SPI-1 T3SS structural genes as in mammalian pathogens. However, the verified 14 InvF regulons by qRT-PCR were not homologous to any effector proteins in pathogenic bacteria. Among the InvF regulons, five InvF regulons had N-terminal signal peptides (Table 3). Specifically, C0J56_14275 encodes an outer membrane protein of the Fap system which might be involved in the export of amyloid subunits for construction of a biofilm matrix (Rouse et al., 2017). C0J56_20550 is a homolog of bacteriocin which might be associated with antagonism against plant pathogens.

To determine the biological functions of SPI-1 T3SS, strains 2P24, 2P24Δ*hilA*, 2P24Δ*invF*, and 2P24Δ*invE-C* were detected in antagonism, motility, chemotactic response to glucose, and biofilm formation. Only 2P24Δ*invF* reduced resistance to *F. graminearum* significantly. In addition, SPI-1 T3SS was reported to be involved in the interaction of bacteria- protozoa. For instance, SPI-1 T3SS of *P. kilonensis* F113 was involved in the resistance to amoeboid grazing (Barret et al., 2013). Whether SPI-1 T3SS of strain 2P24 participates in the resistance to amoeba and nematodes in the soil is worthy of subsequent investigation.

2P24Δ*hilA*, 2P24Δ*invF*, and 2P24Δ*invE-C* reduced the chemotactic responses to glucose significantly. Production of bacterial chemotaxis relies on membrane surface chemical-recognition receptors, intracellular signaling systems, and the movement of flagella (Li and Mu, 2006). In this study, we found that SPI-1 T3SS was not associated with motility of strain 2P24. It is possible that the influence of SPI-1 mutants on the glucose chemotactic response may be due to the following reasons: (i) SPI-1 acted as a sensory receptor for glucose in the external environment, and the absence of the SPI-1 T3SS device reduced the sensitivity to glucose, which affected the chemotactic response to glucose; and (ii) transcriptional factors HilA and InvF mutants affected the expression of chemotactic sensing receptors or chemotactic signal-transduction related proteins.

Type III secretion system was recognized redundant with flagella in mammalian and plant pathogens due to their similarities in the secretion systems that serve flagellar biogenesis and type III effector delivery (Miao et al., 2006; Wei et al., 2013). SPI-1 T3SS of *Salmonella* secreted and translocated flagellin into host cytoplasm, which activated a potent host-defense pathway (Miao et al., 2006). Our results showed that mutation of the SPI-1 T3SS in strain 2P24 reduced the flagellin-triggered ROS burst in planta. This result suggests that the flagellin might be secreted through the SPI-1 T3SS in strain 2P24. Loss of SPI-1 T3SS reduced flagellin output, which led to a decline in ROS burst.

## Data Availability Statement

The datasets presented in this study can be found in online repositories. The names of the repository/repositories and accession number(s) can be found in the article/Supplementary Material.

## Author Contributions

H-LW designed the research. JW, YL, and YG performed the research. JW and H-LW analyzed the data and wrote the manuscript. All authors contributed to the article and approved the submitted version.

## Conflict of Interest

The authors declare that the research was conducted in the absence of any commercial or financial relationships that could be construed as a potential conflict of interest.

## Publisher’s Note

All claims expressed in this article are solely those of the authors and do not necessarily represent those of their affiliated organizations, or those of the publisher, the editors and the reviewers. Any product that may be evaluated in this article, or claim that may be made by its manufacturer, is not guaranteed or endorsed by the publisher.

## Footnotes

1
https://meme-suite.org/meme/tools/fimo

## Supplementary Material

Supplementary Figure 1 KEGG pathway analyses of DEGs of 2P24Δ*invF* that compared with strain 2P24 at 6 h **(A)** and 12 h **(B)** after being incubated in MG medium.

## References

[B1] Alavi S. M. Sanjari S. Durand F. Brin C. Manceau C. Poussier S. (2008). Assessment of the genetic diversity of *Xanthomonas axonopodis* pv. phaseoli and *Xanthomonas fuscans* subsp. fuscans as a basis to identify putative pathogenicity genes and a type III secretion system of the SPI-1 family by multiple suppression subtractive hybridizations. *Appl. Environ. Microbiol.* 74 3295–3301. 10.1128/AEM.02507-07 18359831 PMC2394934

[B2] Barret M. Egan F. Moynihan J. Morrissey J. P. Lesouhaitier O. O’Gara F. (2013). Characterization of the SPI-1 and RSP type three secretion systems in *Pseudomonas* fluorescens F113. *Environ. Microbiol. Rep.* 5 377–386. 10.1111/1758-2229.12039 23754718

[B3] Büttner D. (2012). Protein export according to schedule: architecture, assembly and regulation of type III secretion systems from plant and animal pathogenic bacteria. *Microbiol. Mol. Biol. Rev.* 76 262–310. 22688814 10.1128/MMBR.05017-11 PMC3372255

[B4] Chauhan A. Layton A. C. Williams D. E. Smartt A. E. Ripp S. Karpinets T. V. (2011). Draft genome sequence of the polycyclic aromatic hydrocarbon-degrading, genetically engineered bioluminescent bioreporter *Pseudomonas* fluorescens HK44. *J. Bacteriol.* 193 5009–5010. 10.1128/JB.05530-11 21742869 PMC3165646

[B5] Chet I. Mitchell R. (1976). Ecological aspects of microbial chemotactic behavior. *Ann. Rev. Microbiol.* 30 221–239. 10.1146/annurev.mi.30.100176.001253 791067

[B6] Christensen D. G. Simpson W. A. Bisno A. L. Beachey E. H. (1982). Adherence of slime-producing strains of Staphylococcus epidermidis to smooth surfaces. *Infect. Immun.* 37 318–326. 6179880 10.1128/iai.37.1.318-326.1982 PMC347529

[B7] Compeau G. Al-Achi B. J. Platsouka E. Levy S.B. (1988). Survival of rifampin-resistant mutants of *Pseudomonas fluorescens* and *Pseudomonas putida* in soil systems. *Appl. Environ. Microbiol.* 54 2432–2438. 10.1128/AEM.54.10.2432-2438.1988 3144244 PMC204279

[B8] Correa V. R. Majerczak D. R. Ammar el D. Merighi M. Pratt R. C. Hogenhout S. A. (2012). The bacterium *Pantoea stewartii* uses two different type III secretion systems to colonize its plant host and insect vector. *Appl. Environ. Microbiol.* 78 6327–6336. 10.1128/AEM.00892-12 22773631 PMC3416588

[B9] Darwin K. H. Miller V. L. (1999). InvF is required for expression of genes encoding proteins secreted by the SPI1 type III secretion apparatus in *Salmonella* typhimurium. *J. Bacteriol.* 181 4949–4954. 10438766 10.1128/jb.181.16.4949-4954.1999 PMC93983

[B10] Darwin K. H. Miller V. L. (2001). Type III secretion chaperone-dependent regulation: activation of virulence genes by SicA and InvF in *Salmonella* typhimurium. *EMBO J.* 20 1850–1862. 10.1093/emboj/20.8.1850 11296219 PMC125432

[B11] Deng W. Marshall N. C. Rowland J. L. McCoy J. M. Worrall L. J. Santos A. S. (2017). Assembly, structure, function and regulation of type III secretion systems. *Nat. Rev. Microbiol.* 15 323–337. 10.1038/nrmicro.2017.20 28392566

[B12] Ding L. Wang Y. (2009). Relationship between flagella-dependent motility and biofilm in bacteria. *Acta Microbiol. Sin.* 49 417–422. 10.3321/j.issn:0001-6209.2009.04.002 19621626

[B13] Egan F. Barret M. O’Gara F. (2014). The SPI-1-like Type III secretion system: more roles than you think. *Front. Plant Sci.* 5:34. 10.3389/fpls.2014.00034 24575107 PMC3921676

[B14] Ellermeier J. R. Slauch J. M. (2007). Adaptation to the host environment: regulation of the SPI1 type III secretion system in *salmonella enterica* serovar typhimurium. *Curr. Opin. Microbiol.* 10 24–29. 10.1016/j.mib.2006.12.002 17208038

[B15] Figurski D. Helinski D. R. (1979). Replication of an origin-containing derivative of plasmid RK2 dependent on a plasmid function provided in trans. *Proc. Natl. Acad. Sci. U.S.A.* 76 1648–1652. 10.1073/pnas.76.4.1648 377280 PMC383447

[B16] Grant C. E. Bailey T. L. Noble W. S. (2011). Fimo: scanning for occurrences of a given motif. *Bioinformatics* 27 1017–1018. 10.1093/bioinformatics/btr064 21330290 PMC3065696

[B17] Gu Y. Wang J. Xia Z. Wei H. L. (2020). Characterization of a versatile plant growth-promoting rhizobacterium *Pseudomonas* mediterranea strain S58. *Microorganisms* 8:334. 10.3390/microorganisms8030334 32120878 PMC7143339

[B18] Henrichsen J. (1972). Bacterial surface translocation: a survey and a classification. *Bacteriol. Rev.* 36 478–503. 10.1128/MMBR.36.4.478-503.1972 4631369 PMC408329

[B19] Jackson R. W. Preston G. M. Rainey P. B. (2005). Genetic characterization of *Pseudomonas* fluorescens SBW25 rsp gene expression in the phytosphere and in vitro. *J. Bacteriol.* 187 8477–8488. 10.1128/JB.187.24.8477-8488.2005 16321952 PMC1317024

[B20] Kimbrel J. A. Givan S. A. Halgren A. B. Creason A. L. Mills D. I. Banowetz G. M. (2010). An improved, high-quality draft genome sequence of the germination-arrest factor-producing *Pseudomonas* fluorescens WH6. *BMC Genomics* 11:522. 10.1186/1471-2164-11-522 20920191 PMC2997014

[B21] Kirzinger M. Butz C. J. Stavrinides J. (2015). Inheritance of pantoea type III secretion systems through both vertical and horizontal transfer. *Mol. Genet. Genomics* 290 2075–2088. 10.1007/s00438-015-1062-2 25982743

[B22] Kumar S. Stecher G. Tamura K. (2016). MEGA7: molecular evolutionary genetics analysis version 7.0 for bigger datasets. *Mol. Biol. Evol.* 7:1870. 10.1093/molbev/msw054 27004904 PMC8210823

[B23] Li Y. Mu B. (2006). Progress in chemotaxis of bacteria. *Chin. J. Appl. Environ. Biol.* 12 135–139. 10.3321/j.issn:1006-687X.2006.01.033 30704229

[B24] Liu P. Wei Z. Zhang L. Q. Liu X. Wei H. L. (2016). Supramolecular structure and functional analysis of the type III secretion system in *Pseudomonas* fluorescens 2P24. *Front. Plant Sci.* 6:1190. 10.3389/fpls.2015.01190 26779224 PMC4700148

[B25] Loper J. E. Hassan K. A. Mavrodi D. V. Davis E. W. Lim C. K. Shaffer B. T. (2012). Comparative genomics of plant-associated *Pseudomonas* spp.: insights into diversity and inheritance of traits involved in multitrophic interactions. *PLoS Genet.* 8:e1002784. 10.1371/journal.pgen.1002784 22792073 PMC3390384

[B26] Marchi M. Boutin M. Gazengel K. Rispe C. Gauthier J. P. Guillerm-Erckelboudt A. Y. (2013). Genomic analysis of the biocontrol strain *Pseudomonas* fluorescens Pf29Arp with evidence of T3SS and T6SS gene expression on plant roots. *Environ. Microbiol. Rep.* 5 393–403. 10.1111/1758-2229.12048 23754720

[B27] Marguerettaz M. Pieretti I. Gayral P. Puig J. Brin C. Cociancich S. (2011). Genomic and evolutionary features of the SPI-1 type III secretion system that is present in *Xanthomonas albilineans* but is not essential for xylem colonization and symptom development of sugarcane leaf scald. *Mol. Plant Microbe Interact.* 24 246–259. 10.1094/MPMI-08-10-0188 20955079

[B28] Mazumder R. Phelps T. J. Krieg N. R. Benoit R. E. (1999). Determining chemotactic responses by two subsurface microaerophiles using a simplified capillary assay method. *J. Microbiol. Methods* 37 255–263. 10.1016/j.scriptamat.2010.02.026 10480269

[B29] Miao E. A. Alpuche-Aranda C. M. Dors M. Clark A. E. Bader M. W. Miller S. I. (2006). Cytoplasmic flagellin activates caspase-1 and secretion of interleukin 1β via Ipaf. *Nat. Immunol.* 7 569–575. 10.1038/ni1344 16648853

[B30] Nazir R. Mazurier S. Yang P. Lemanceau P. Van Elsas J. D. (2017). The ecological role of type three secretion systems in the interaction of bacteria with fungi in soil and related habitats is diverse and context-dependent. *Front. Microbiol.* 8:38. 10.3389/fmicb.2017.00038 28197129 PMC5282467

[B31] Pallen M. J. Beatson S. A. Bailey C. M. (2005). Bioinformatics, genomics and evolution of non-flagellar type-III secretion systems: a darwinian perspective. *FEMS Microbiol. Rev.* 29 201–229. 10.1016/j.femsre.2005.01.001 15808742

[B32] Preston G. M. Bertrand N. Rainey P. B. (2001). Type III secretion in plant growth-promoting *Pseudomonas* fluorescens SBW25. *Mol. Microbiol.* 41 999–1014. 10.1046/j.1365-2958.2001.02560.x 11555282

[B33] Rashid M. H. Kornberg A. (2000). Inorganic polyphosphate is needed for swimming, swarming, and twitching motilities of *Pseudomonas aeruginosa*. *Proc. Natl. Acad. Sci. U.S.A.* 97 4885–4890. 10.1073/pnas.060030097 10758151 PMC18327

[B34] Redondo-Nieto M. Barret M. Morrissey J. Germaine K. Martinez-Granero F. Barahona E. (2013). Genome sequence reveals that *Pseudomonas* fluorescens F113 possesses a large and diverse array of systems for rhizosphere function and host interaction. *BMC Genomics* 14:54. 10.1186/1471-2164-14-54 23350846 PMC3570484

[B35] Rouse S. L. Hawthorne W. J. Berry J. L. Chorev D. S. Ionescu S. A. Lambert S. (2017). A new class of hybrid secretion system is employed in *Pseudomonas* amyloid biogenesis. *Nat. Commun.* 8:263. 10.1038/s41467-017-00361-6 28811582 PMC5557850

[B36] Sambrook E. Fritsch F. Maniatis T. (1989). Molecular cloning. Cold spring harbor press, cold spring harbor. *Am. J. Hum. Genet.* 73 1162–1169.

[B37] Stringlis I. A. Zamioudis C. Berendsen R. L. Bakker P. A. H. M. Pieterse C. M. J. (2019). Type III secretion system of beneficial rhizobacteria *Pseudomonas* simiae WCS417 and *Pseudomonas* defensor WCS374. *Front. Microbiol.* 10:1631. 10.3389/fmicb.2019.01631 31379783 PMC6647874

[B38] Triplett L. R. Zhao Y. Sundin G. W. (2006). Genetic differences between blight-causing *Erwinia* species with differing host specificities, identified by suppression subtractive hybridization. *Appl. Environ. Microbiol.* 72 7359–7364. 10.1128/AEM.01159-06 16963554 PMC1636173

[B39] Troisfontaines P. Cornelis G. R. (2005). Type III secretion: more systems than you think. *Physiology* 20 326–339. 10.1152/physiol.00011.2005 16174872

[B40] Ugochukwu U. C. Jones M. D. Head I. M. Manning D. A. Fialips C. I. (2013). Compositional changes of crude oil SARA fractions due to biodegradation and adsorption on colloidal support such as clays using Iatroscan. *Environ. Sci. Pollut. Res.* 20 6445–6454. 10.1007/s11356-013-1635-8 23589240

[B41] Viollet A. Pivato B. Mougel C. Cleyet-Marel J. C. Gubry-Rangin C. Lemanceau P. (2017). *Pseudomonas* fluorescens C7R12 type III secretion system impacts mycorrhization of *Medicago truncatula* and associated microbial communities. *Mycorrhiza* 27 23–33. 10.1007/s00572-016-0730-3 27549437

[B42] Wei H. L. Chakravarthy S. Worley J. N. Collmer A. (2013). Consequences of flagellin export through the type III secretion system of *Pseudomonas* syringae reveal a major difference in the innate immune systems of mammals and the model plant *Nicotiana benthamiana*. *Cell. Microbiol.* 15 601–618. 10.1111/cmi.12059 23107228

[B43] Wei H. L. Wang Y. Zhang L. Q. Tang W. H. (2004a). Identification and characterization of biocontrol bacterial strain 2P24 and CPF-10. *Acta Phytopathol. Sin.* 34 80–85.

[B44] Wei H. L. Zhou H. Y. Zhang L. Q. Wang Y. Tang W. H. (2004b). Experimental evidence on the functional agent of 2,4-diacetylphloroglucinolin biocontrol activity of *Pseudomonas* fluorescens 2P24. *Acta Microbiol. Sin.* 44 663–666. 10.13343/j.cnki.wsxb.2004.05.024

