# Supplementary figures and images for: Characterization of the SPI-1 Type III Secretion System in Pseudomonas fluorescens 2P24
Source: Front Microbiol. 2021 Sep 21;12:749037. doi: 10.3389/fmicb.2021.749037 (PMC8490769; doi:10.3389/fmicb.2021.749037)

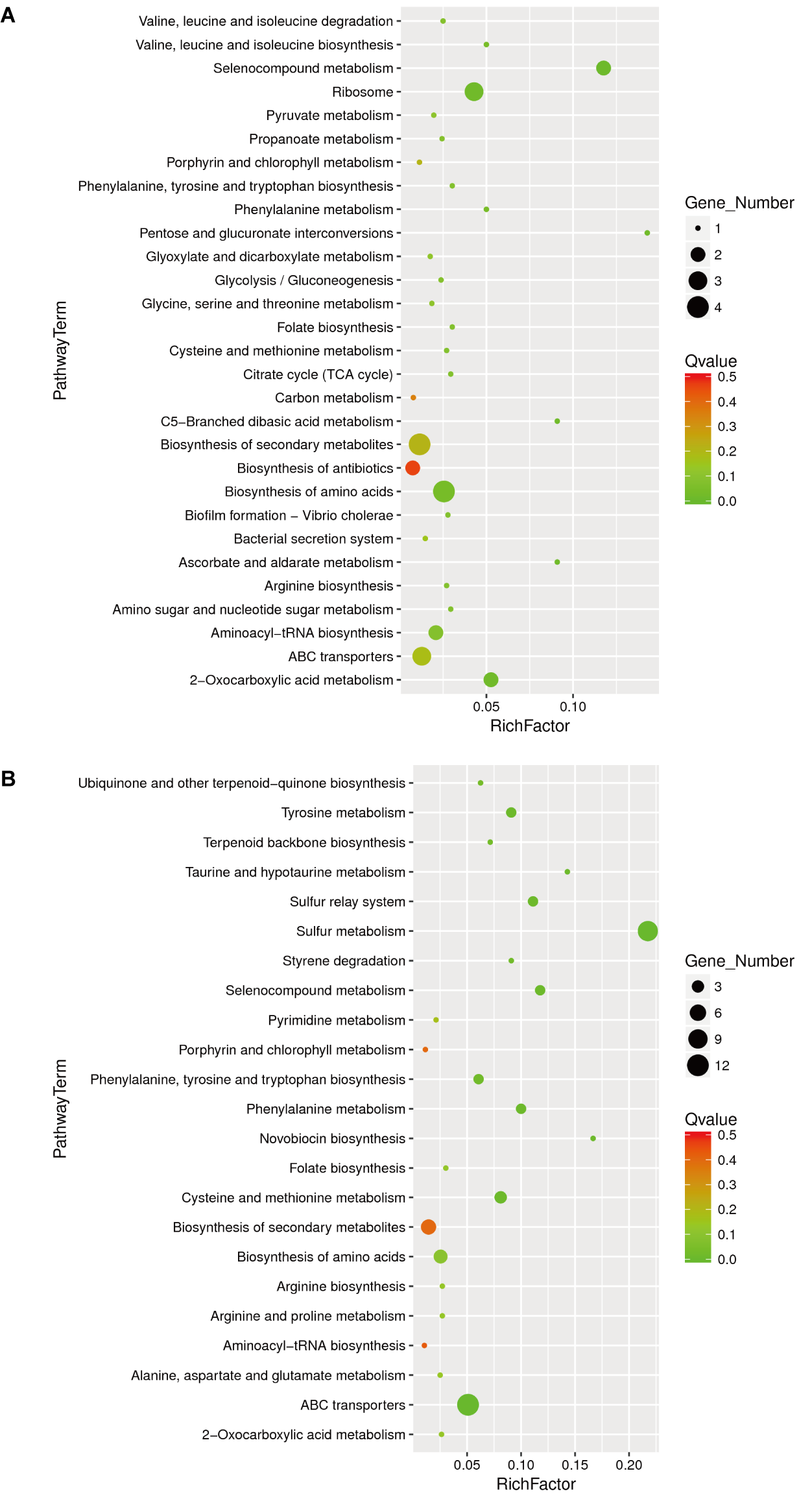

Supplement: Supplementary Figure 1 — KEGG pathway analyses of DEGs of 2P24ΔinvF that compared with strain 2P24 at 6 h (A) and 12 h (B) after being incubated in MG medium. [file Image_1.tif]
